# Evaluation of the HB&L System for the Microbiological Screening of Storage Medium for Organ-Cultured Corneas

**DOI:** 10.1155/2013/670947

**Published:** 2013-08-28

**Authors:** D. Camposampiero, S. Grandesso, E. Zanetti, S. Mazzucato, M. Solinas, M. Parekh, A. C. Frigo, M. Gion, D. Ponzin

**Affiliations:** ^1^The Veneto Eye Bank Foundation, Via Paccagnella 11, 30174 Venice, Italy; ^2^Unità Operativa Complessa Laboratorio Analisi Chimico-Cliniche e Microbiologiche, Ospedale dell' Angelo, 30174 Venezia, Italy; ^3^Università degli Studi di Padova, Dipartimento di Scienze Cardiologiche, Toraciche e Vascolari, Via Giustiniani, 2-35128 Padova, Italy

## Abstract

*Aims*. To compare HB&L and BACTEC systems for detecting the microorganisms contaminating the corneal storage liquid preserved at 31°C. *Methods*. Human donor corneas were stored at 4°C followed by preservation at 31°C. Samples of the storage medium were inoculated in BACTEC Peds Plus/F (aerobic microorganisms), BACTEC Plus Anaerobic/F (anaerobic microorganisms), and HB&L bottles. The tests were performed (a) after six days of storage, (b) end of storage, and (c) after 24 hours of preservation in deturgescent liquid sequentially. 10,655 storage and deturgescent media samples were subjected to microbiological control using BACTEC (6-day incubation) and HB&L (24-hour incubation) systems simultaneously. BACTEC positive/negative refers to both/either aerobic and anaerobic positives/negatives, whereas HB&L can only detect the aerobic microbes, and therefore the positives/negatives depend on the presence/absence of aerobic microorganisms. *Results*. 147 (1.38%) samples were identified positive with at least one of the two methods. 127 samples (134 identified microorganisms) were positive with both HB&L and BACTEC. 14 HB&L+/BACTEC− and 6 BACTEC+/HB&L− were identified. Sensitivity (95.5%), specificity (99.8%), and positive (90.1%) and negative predictive values (99.9%) were high with HB&L considering a 3.5% annual contamination rate. *Conclusion*. HB&L is a rapid system for detecting microorganisms in corneal storage medium in addition to the existing methods.

## 1. Introduction

Endophthalmitis is a microbial infection within the eye that results in severe inflammation. It is a severe complication of intraocular surgeries like penetrating keratoplasty (PK) or cataract surgery where the risk of bacterial or fungal infection increases with transplantation of a contaminated cornea [1, 2]. It has been shown that the microorganisms responsible for the postoperative endophthalmitis are usually derived from the transplanted corneas; however, involvement of other postoperative risk factors for ocular infection has also been determined [3].

The growth of bacterial strains susceptible to the antibiotics (typically penicillin and streptomycin) and antifungal substances (amphotericin B) added to the preservation medium allows to determine the microbial growth and eventually discard the contaminated cornea. The presence of bacteria is not always indicated by the turbidity or the yellowish appearance of the culture medium; therefore, both the efficacy and sensitivity of the detection method of microbial contamination become a critical factor. Moreover, if long-term organ culture is compared to short-term hypothermic preservation (usually between 2°C and 8°C), organ culture appears to be more safe due to the length of culture period (7–30 days) and the temperature (typically 31°C–37°C) which allows the growth and therefore detection of the microorganisms [4, 5].

Conventional microbiological controls are currently performed using standard bacteriological media in aerobic and anaerobic environments whereas Sabouraud broth is a routine medium for detection of fungi [6]. Other options include use of Bactec blood bottles (Becton Dickinson, USA) incubated in the Bactec instrument (based on the detection of CO_2_ produced by microorganisms) which offer many advantages over the standard microbiological techniques [7–9]. This system has a minimal risk of contamination caused due to handling, better detection with low bacterial inoculum, and major promotion of microbial growth thanks to the presence of antimicrobial removal device (ARD) and is a rapid detection method for the continuous monitoring of bacterial growth (every ten minutes). To meet the needs of microbiological safety of corneas distributed for transplantation, a test that can rapidly report the presence of microorganisms in the medium during the storage of tissue is recommended.

The HB&L system (Alifax, Padua, Italy) is a rapid and automated method of bacterial screening for urine and biological samples. It uses light scattering technology to detect the growth of bacteria. Recently, utilisation of HB&L for the culture of fluid samples has been reported [10]. 

This paper describes a comparative study between the performances of the HB&L and the Bactec system, “in house” validated [11] and used in The Veneto Eye Bank Foundation (FBOV) laboratory to identify the microorganisms that contaminate the corneal culture medium.

## 2. Materials and Methods

### 2.1. Tissue Selection and Preservation

The investigation involved all of the corneal tissues and ocular globes received by the FBOV eye bank between June 2010 and August 2011. During this period 5,156 ocular tissues were processed (4,264 corneas and 892 eye globes), out of which 322 were discarded at the first control (unsuitable for transplantation due to poor biological quality). 10,655 storage liquid samples were analyzed for the remaining samples. 4,834 samples were analyzed with storage medium “S” (after six days of storage), 2,924 at the end of storage time “DT,” and 2,897 from the deswelling medium “T” (after 24 hours of preservation of the cornea in deturgescent liquid). All corneas were serially tested in different phases of preservation (from S to DT to T). Those corneas with positive contamination were discarded at the respective step.

Human corneoscleral rims were obtained within 24 hours after the donor's death. Before the recovery of the tissues, they were pretreated with 0.5% (wt/vol) povidone-iodine for 2 minutes, washed with a sterile saline solution, and preserved at 4°C. The corneoscleral rims were processed and manipulated under the aseptic conditions. The conjunctival remnants were excised before preservation. The tissues were rinsed with sterile 0.15 M sodium chloride solution and were preserved in a polycarbonate bottle containing 100 mL of sterilized culture medium which includes streptomycin as a protective against fungi and Gram positive and Gram negative bacteria. The tissues were further suspended by a device plucked to the scleral rim which helps the tissue to float. At the end of the cornea culture, the endothelium was exposed to 0.25% (wt/vol) trypan blue stain to count the nuclei of nonviable endothelial cells using 1.4% (wt/vol) sucrose solution (hypotonic) for clear visualization of the cellular borders and endothelial cell density. Both trypan blue solution and the hypotonic sucrose solution used for the evaluation of the endothelial quality were sterilized and tested for bacterial contaminations. Later, the corneas were immersed in the deswelling medium to restore the normal thickness and were finally delivered to the surgeons. The deswelling medium contains 6% (wt/vol) dextran-T500 in addition to the culture medium.

In case of globe retrieval, the cornea was isolated in the laboratory inside a laminar air flow hood and stored at 31°C as described earlier.

### 2.2. Microbiological Techniques

The microbiological tests were performed by inoculating a sample of the culture medium in Bactec Peds Plus/F bottles and Bactec Plus Anaerobic/F bottles after six days of preservation (S), at the end of preservation (DT), and after 24 hours of intransient phase in deturgescent medium (T). For this study, all samples of preservation and deturgescent medium were subjected to microbiological controls using the Bactec and the HB&L systems simultaneously. Bactec was considered positive if it showed positive results for either aerobic or anaerobic; similarly, aerobic/anaerobic negative results were referred to as Bactec negative.

0.5 mL of storage medium was inoculated from the same sample in HB&L bottle and incubated for 24 hrs, according to the manufacturer's instructions, in order to detect the minimal bacterial presence (<50 CFU/mL).

For all cultured corneas, first microbiological test was performed after 6 days of preservation by collecting a sample of the storage medium (inoculum). The samples of medium were inoculated (3 mL for the Peds Plus test and 3 mL for the anaerobic test) in two bottles and placed in a Bactec 9240 automat. 0.5 mL of storage medium was inoculated from the same sample in HB&L bottle and incubated for 24 hrs according to the manufacturer's instructions in order to detect the minimal bacterial presence (<50 CFU/mL).

The last microbiological test on the storage medium (DT) was performed on the last day of culture (mean = 21.5, standard deviation = 8.1 days); after DT, the corneas were transferred to the deswelling medium according to the conventional protocol used for organ culture.

For the corneas that were placed in the deswelling medium, the microbiological test included inoculating 3 mL of the media sample in Bactec Peds/F Plus only and 0.5 mL in HB&L, 24 hours after the transfer of the corneas.

All samples were inoculated simultaneously and incubated for 24 hours in HB&L and for 6 days in Bactec system. Isolates were identified with the standard bacteriological techniques that are routinely used. Isolated bacteria were not tested for common resistance to the antibiotics since the microbial growth is sufficient to exclude the cornea from transplantation.

### 2.3. Statistical Analysis

STARD (Standards for Reporting of Diagnostic Accuracy) guidelines for new assays were followed for this experiment [12]. BACTEC system is an internal validated method prescribed by the in house standard operating procedure; it was therefore considered as gold standard for this study.

Sensitivity (Se) and specificity (Sp) of HB&L were estimated at 95% confidence interval (95% CI) with the binomial exact method [13]. Positive (PPV) and negative (NPV) predictive values were estimated considering a mean annual contamination rate of 3.5% (taking 6 years of FBOV activity into account).

The likelihood ratios (LR) were also calculated for this study. The positive LR (LR+) indicated that the frequency of a positive result with HB&L system was more likely to be observed in specimens positive with the BACTEC system than in those that were negative. The negative LR (LR−) indicated that the frequency of a negative result with HB&L system was more likely to be observed in specimens negative with the BACTEC system than in those with a positive result. The test being evaluated was more accurate if the LR differed by 1. LR+ above 10 and LR− below 0.1 were considered as convincing diagnostic lines of evidence [14]. Confidence interval at 95% level for positive and negative likelihood ratios was calculated with the method proposed by Simel and colleagues [15].

## 3. Results

10,655 samples were analyzed, out of which 4,834 were “S,” 2,924 “DT,” and 2,897 “T.” 147 microbiological tests were positive (147/10,655) with an overall contamination rate of 1.38%. This rate was 2.62% (127/4,834) for S, 0.51% (15/2,924) for DT, and 0.17% (5/2,897) for T.

127 positive samples with both HB&L and BACTEC systems (86.4% of total positive; 1.2% of all analyzed samples) were found with the occurrence of at least one isolated microorganism. In seven of the 127 samples, two microorganisms were found simultaneously in both detection systems. Moreover, 6 samples were found positive only with BACTEC system (4.1% of total positive; 0.1% of all analyzed samples) and 14 positive samples only with HB&L (9.5% of total positive; 0.1% of all analyzed samples) (Table 1). Sensitivity and specificity of HB&L were recorded as 95.5% (95% CI: 90.4%–98.3%) and 99.9% (95% CI: 99.8%–99.9%), respectively, with consequent high PPV of 96.2% (95% CI: 93.7%–97.9%) and NPV of 99.8% (95% CI: 99.7%–99.9%). The positive and negative likelihood ratios resulted in 717.7 (424.6–1212.9) and 0.05 (0.02–0.10), respectively (Table 2).

All of the 14 HB&L+/Bactec− samples found in storage medium were probably due to manipulation error during the sample inoculation. The isolated pathogens detected using two different systems are listed in Table 1, whereas the microorganisms that were isolated in different media are listed in Table 3.

In the cases where the microbial growth was observed in T step, the same microorganism was detected in DT and S, whereas when we had positive result in the DT phase, the same microorganism was detected in the S phase.

The mean detection time of 33.5 hours was found between inoculation and positivity of aerobic and anaerobic Bactec bottles (min 15.7 hrs, max 126.3 hrs, and median 41.8 hrs) and the mean detection time of 4.2 hrs for the HB&L system with min 0.7 hrs, max 17.8 hrs, and median 8.6 hrs.

In particular, by the end of the maximum incubation time of HB&L (24 hrs), 18/127 (14.2%) positive samples with Bactec and HB&L systems were found. Among the positive ones, 17/127 (13.4%) belonged to S and 1/127 (0.79%) to DT. None of them resulted positive amongst the T group.

## 4. Discussion

It is important to have a standardized protocol that allows the microbial detection as quickly as possible before transplantation for the eye banks that are involved in the distribution of organ-cultured corneas. In order to reach a high level of microbial safety, FBOV has adopted the Bactec system since 2002 for all corneas that are preserved at 31°C [11]. According to the FBOV SOP (standard operating protocol), the BACTEC system requires a 6-day protocol for the final negative result to occur; the last tests (DT and T tests) are usually under evaluation and coincide with the surgery (usually between 2 and 7 days after the corneas are transferred to the deswelling medium). This could result in microbiologically positive corneas used for surgery, with an associated risk of causing a corneal infection in the recipient eye. Considering the recent utilization of HB&L system for the culture of fluid samples [10], we compared these results with the ones obtained using Bactec system in order to introduce the HB&L system in our microbiological screening.

Considering the theoretical sensitivity of the HB&L system [16], we found that the incubation period should be longer (24 hrs) than the one applied in other procedures in order to reduce the minimal detectable quantity of microorganisms. We observed that BACTEC+/HB&L+ samples indicated the presence of bacteria in the cornea because the isolated organisms were the same in the two diagnostic systems. If samples show BACTEC−/HB&L+, then it should be considered as contamination of the sample inoculums. It was also found that staphylococci (the only microorganisms identified) were not found in the preserving liquid analyzed with traditional culture. All of these results were found during the initial use of HB&L system when the personnel were not completely trained, bound to human error, and therefore less accurate with the system. In fact, the inoculum area of the HB&L bottles is very small, and therefore it is important that the involved personnel should be well trained to reduce the error and increase the efficacy. 

The only exception was the detection of one *Candida albicans *positive sample which was found in HB&L and in the preserving fluid which did not appear in Bactec bottles. The six samples that resulted in BACTEC+/HB&L− were positive for filamentous fungi (three *Fusarium *spp. and one *Aspergillus fumigatus*) and two for anaerobic bacteria (*Bacteroides thetaiotaomicron*); this was an expected limitation of the HB&L system because the broth is suitable only for aerobic bacteria and not for filamentous fungi and anaerobic bacteria. We chose a one-day protocol for HB&L system in order to have a rapid response knowing that filamentous fungi do not grow in the used broth even if incubated for a longer period.

Another aspect to be considered is the presence of antimicrobial removal device (ARD) in the BACTEC bottles. The recognized importance of ARD in order to have the optimal microbial growth was not demonstrated; in fact, the BACTEC+/HB&L− results were represented only by anaerobic bacteria and filamentous fungi. If the ARD had any influence on the microorganism detection, we would have found more aerobic and antibiotic susceptible bacteria in BACTEC system but not in the HB&L system.

Overall the HB&L system showed an excellent diagnostic performance as compared to the standard culture in BACTEC system in terms of sensitivity, specificity, and LR+ and LR−. Therefore, it is essential that a negative HB&L result in 24 hrs is confirmed by a six-day BACTEC protocol which is also the conventional method used by FBOV. Using this method we were able to reduce the time required to find a negative result and to have a microbiologically safe tissue. This aspect is confirmed by the high specificity value (99.8%) and even more by the higher negative predictive value (99.9%). Furthermore, the values of positive and negative likelihood ratios indicate a good capacity of the HB&L to both rule in and rule out the presence of contamination.

Concerning the positive samples, it is important to have the result as soon as possible. In our experience and according to Reisner and Woods [17], the HB&L system seemed quicker than the BACTEC system in revealing all of the microbial growth. None of the samples interfered with the light scattering of the HB&L system that is, no positive samples were found due to the presence of residual particles in suspension (e.g., epithelial cells).

In conclusion, we intend to further investigate new broths that are under development which are specifically formulated for anaerobic bacteria and fungi. Thus, with the results and our experience, the HB&L system can be proposed for a faster detection of microorganisms in the storage medium for corneas as compared to the routinely used method. However, due to its limitation in detecting filamentous fungi and anaerobic bacteria, it is recommended to be used in association or combination with a wide spectrum detection system.

## Figures and Tables

**Table 1:** Isolated pathogens in comparative study between BACTEC and HB&L methods.

	HB&L+	HB&L−
BACTEC+	*Staphylococcus* spp. (46) *Enterococcus* spp. (22) *Streptococcus* spp. (3) *Kocuria kristinae* (4) *Pseudomonas aeruginosa* (6) *Acinetobacter baumannii* (5) *Escherichia* coli (4) *Stenotrophomonas maltophilia* (4) *Proteus mirabilis* (2) *Achromobacter xylosoxidans* (1) *Brevundimonas diminuta* (2) *Candida* spp. (33) *Saccharomyces cerevisiae* (1) *Trichosporum asahii* (1) Total: 134 microorganisms (in 127 samples)	*Fusarium* spp. (3) *Bacteroides thetaiotaomicron* (2) *Aspergillus fumigatus* (1) Total: 6 microorganisms (in 6 samples)

BACTEC−	*Staphylococcus* spp. (13) *Candida albicans* (1) Total: 14 microorganisms (in 13 samples)	Total: 10,508

**Table 2:** Sensitivity, specificity, predictive values and likelihood ratios.

Parameters	Formula	Value (95% CI)
Sensitivity (Se) (%)	$\frac{\text{TP}}{\left(\text{TP}+\text{FN}\right)}$	95.5 (90.4–98.3)
Specificity (Sp) (%)	$\frac{\text{TN}}{\left(\text{TN}+\text{FP}\right)}$	99.9 (99.8–99.9)
Positive predictive value (PPV) (%)	$\frac{\text{Se}\times \text{C}}{\text{Se}\times \text{C}+\left(1-\text{Sp}\right)\times \left(1-\text{C}\right)}$	96.2 (93.7–97.9)
Negative predictive value (NPV) (%)	$\frac{\text{Sp}\times (1-\text{C})}{\text{Sp}\times \left(1-\text{C}\right)+\left(1-\text{Se}\right)\times \text{C}}$	99.8 (99.7–99.9)
Positive likelihood ratio (LR+)	$\frac{\text{Se}}{\text{1}-\text{Sp}}$	717.7 (424.6–1212.9)
Negative likelihood ratio (LR−)	$\frac{1-\text{Se}}{\text{Sp}}$	0.05 (0.02–0.10)

TP: true positive; FN: false negative; FP: false positive; C: prevalence.

**Table 3:** Microorganisms isolated in different media.

Microorganisms	S	DT	T
*Staphylococcus haemolyticus *	39*	2	1
*Staphylococcus epidermidis *	8	—	—
*Staphylococcus aureus *	7**	—	—
*Staphylococcus cohnii *	2	—	—
*Enterococcus faecium *	12***	—	—
*Enterococcus faecalis *	7	—	—
*Enterococcus gallinarum *	3	—	—
*Streptococcus mitis *	3	—	—
*Kocuria kristinae *	4	—	—
*Pseudomonas aeruginosa *	5	1	—
*Acinetobacter baumannii *	4	1	—
*Escherichia coli *	4	—	—
*Stenotrophomonas maltophilia *	4	—	—
*Proteus mirabilis *	2	—	—
*Brevundimonas diminuta *	2	—	—
*Achromobacter xylosoxidans *	1	—	—
*Bacteroides thetaiotaomicron *	2	—	—
*Candida albicans *	14****	8	2
*Candida glabrata *	3	2	2
*Candida parapsilosis *	2	—	—
*Candida krusei *	—	1	—
*Saccharomyces cerevisiae *	1	—	—
*Trichosporon asahii *	1	—	—
*Fusarium* spp.	3	—	—
*Aspergillus fumigatus *	1	—	—
**Total**	**134**	**15**	**5**

*1 + *C. albicans* and *C. glabrata*; **1 + *C. albicans*; ***1 + *C. albicans*; ****1 + *S. haemolyticus*, *E. faecium*, and *S. aureus*.
